# The role of mitogen-activated protein kinases and sterol receptor coactivator-1 in TGF-β-regulated expression of genes implicated in macrophage cholesterol uptake

**DOI:** 10.1038/srep34368

**Published:** 2016-09-30

**Authors:** Rebecca C. Salter, Pelagia Foka, Thomas S. Davies, Hayley Gallagher, Daryn R. Michael, Tim G. Ashlin, Dipak P. Ramji

**Affiliations:** 1 Cardiff School of Biosciences, Cardiff University, Sir Martin Evans Building, Museum Avenue, Cardiff, CF10 3AX, United Kingdom

## Abstract

The anti-atherogenic cytokine TGF-β inhibits macrophage foam cell formation by suppressing the expression of key genes implicated in the uptake of modified lipoproteins. We have previously shown a critical role for p38 MAPK and JNK in the TGF-β-mediated regulation of apolipoprotein E expression in human monocytes. However, the roles of these two MAPK pathways in the control of expression of key genes involved in the uptake of modified lipoproteins in human macrophages is poorly understood and formed the focus of this study. TGF-β activated both p38 MAPK and JNK, and knockdown of p38 MAPK or c-Jun, a key downstream target of JNK action, demonstrated their requirement in the TGF-β-inhibited expression of several key genes implicated in macrophage lipoprotein uptake. The potential role of c-Jun and specific co-activators in the action of TGF-β was investigated further by studies on the lipoprotein lipase gene. c-Jun did not directly interact with the minimal promoter region containing the TGF-β response elements and a combination of transient transfection and knock down assays revealed an important role for SRC-1. These studies provide novel insights into the mechanisms underlying the TGF-β-mediated inhibition of macrophage gene expression associated with the control of cholesterol homeostasis.

Atherosclerosis is a progressive inflammatory disorder of the vasculature and is one of the leading causes of mortality in western societies^1^. Atherosclerosis is initiated by the activation of the vascular endothelium by a multitude of risk factors including diet, smoking and genetic predisposition^1^. The disease is characterized by the formation of fibrous plaques composed of cholesterol, lipids, inflammatory cells and cellular debris^1^. Such plaques can become unstable and rupture, resulting in thrombosis, myocardial infarction and stroke^1^.

Formation of lipid-laden foam cells is a critical early step in atherosclerosis^1^,^2^. The activation of the endothelium recruits immune cells, including T-lymphocytes and monocytes, to the arterial intima where the latter differentiate into macrophages^1^,^2^. Macrophage differentiation is associated with increased expression of surface scavenger receptors and other enzymes, such as lipoprotein lipase (LPL), that allow the uptake of modified low-density lipoproteins (LDL), which subsequently accumulate in the vessel wall^1^,^2^,^3^. Formation of foam cells is regulated by novel and classical cytokines such as interleukin (IL)-33, tumour necrosis factor-like protein 1A (TL1A), interferon-γ (IFN-γ) and transforming growth factor-β (TGF-β)^1^,^2^,^4^,^5^,^6^,^7^.

TGF-β is widely recognized to have anti-atherogenic actions^1^,^2^,^4^,^8^,^9^. For example, reduced levels of this cytokine have been observed in patients with advanced atherosclerosis and regions of the aorta with low TGF-β expression have a high probability of lesion development^8^,^9^,^10^. Inhibition of TGF-β using neutralizing antibodies or expression of dominant-negative forms of TGF-β receptors results in accelerated lesion development and elevated inflammatory responses in mouse model systems^4^,^8^,^9^,^11^. In addition, TGF-β inhibits foam cell formation as shown by studies in cultured macrophages and in mouse model systems^7^,^8^,^9^,^12^,^13^. Numerous *in vitro* studies from our own and other laboratories have demonstrated that TGF-β negatively regulates the expression of key genes implicated in cholesterol uptake such as LPL and scavenger receptors (SR)–A1, -B1 and CD36^7^,^8^,^9^,^12^,^13^.

TGF-β classically signals through the Smad pathway but activation of other signaling pathways by the cytokine have also been identified^8^,^9^,^14^,^15^. For example, the activation of mitogen-activated protein kinases (MAPKs) by TGF-β has been demonstrated in a number of cell types^8^,^9^,^14^,^15^. However, relatively little is known about the role of MAPKs in TGF-β signaling in macrophages, particularly in relation to the control of expression of key genes implicated in the regulation of cholesterol homeostasis and foam cell formation. Further studies are necessary given the potent anti-foam cell and anti-atherogenic role of this cytokine^8^,^9^. Investigation of the signaling pathways underlying TGF-β actions in atherosclerosis may ultimately contribute to the identification of novel therapeutic targets for the treatment of this disease. Our previous studies on the TGF-β-mediated induction of apoplipoprotein E (apoE) gene expression in human monocytes revealed an important role for c-Jun N-terminal kinase (JNK) and p38 MAPK^16^. As apoE is involved in the control of macrophage cholesterol efflux, the role of these two kinases in the TGF-β-mediated regulation of key genes implicated in the uptake of modified LDL, such as SR-A1, SR-B1, CD36 and LPL, remains to be determined. In addition, as the expression of the apoE gene is induced by TGF-β, the roles of these kinases in inhibition of gene expression by this cytokine remains unclear. The purpose of this study was therefore to investigate the role of these kinases in the TGF-β-mediated inhibition of expression of key genes implicated in the control of modified lipoprotein uptake by human macrophages together with the potential underlying mechanisms.

## Results

### TGF-β activates JNK and p38 MAPK and modulates the expression of key genes implicated in the uptake of modified lipoproteins in human macrophages

Human monocyte-derived THP-1 macrophages are extensively used as a model for investigating macrophage gene expression and cellular function in relation to atherosclerosis because of conserved responses with primary cultures of human monocyte-derived macrophages (HMDM) and *in vivo* models^5^,^6^,^7^,^17^. This system was therefore employed to investigate the potential role of the JNK and p38 MAPK pathways in TGF-β signaling in relation to the expression of key genes implicated in the control of macrophage lipoprotein uptake.

We have previously shown that TGF-β activates JNK and p38 MAPK in THP-1 monocytes^16^. Representative experiments were initially carried out to confirm that TGF-β also activates these two signaling pathways in human macrophages. Western blot analysis showed that TGF-β increased the levels of phosphorylated, activated forms of p38 MAPK and JNK, without affecting the total levels of these proteins, with maximal levels of activated protein typically attained at 24 h (Supplementary Fig. 1A,B). TGF-β also increased the levels of phospho-SEK-1, an upstream activator of JNK (Supplementary Fig. 1C). Representative experiments with p38 MAPK also showed increased levels of phosphorylated, activated protein in HMDM following treatment of the cells with TGF-β (Supplementary Fig. 1D). In order to confirm that the increase in the levels of the phosphorylated proteins were associated with enhanced activities of the enzymes, representative experiments were carried out for JNK using the more sensitive cell-free kinase assays in which the ability of the immunoprecipitated protein to phosphorylate its key downstream substrate, c-Jun, *in vitro* was analysed. These assays confirmed that TGF-β induces JNK activity in THP-1 macrophages (Supplementary Fig. 1E) and primary cultures of HMDM (Supplementary Fig. 1F). Having established that TGF-β activates JNK and p38 MAPK in THP-1 macrophages and HMDM, further studies analyzed the role of these pathways in the regulation of gene expression.

**Figure 1:**
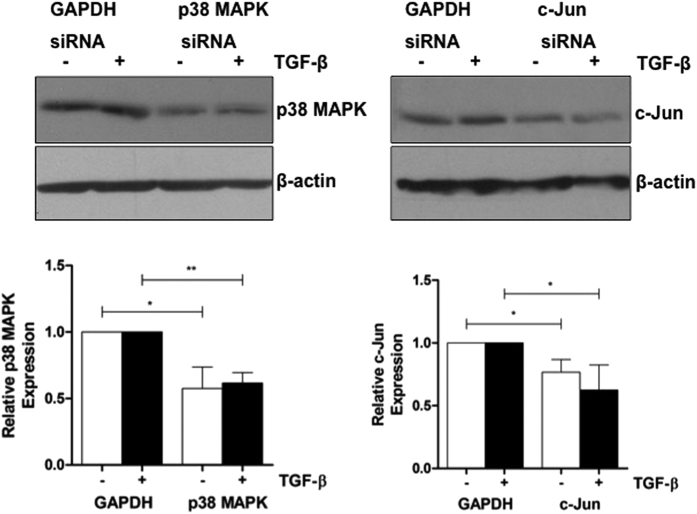
siRNA-mediated knockdown of p38 MAPK or c-Jun in THP-1 macrophages. THP-1 monocytes were transfected with the indicated siRNA, differentiated into macrophage using PMA for 24 h, and then incubated with vehicle (−, empty bars) or TGF-β (30 ng/ml) (+, filled bars) for 24 h as described in Materials and Methods. Equal amounts of protein extracts were subjected to western blot analysis using antisera against p38 MAPK, c-Jun or β-actin. The image shows the signal from the immunoreactive p38 MAPK (43 kDa), c-Jun (39 kDa) or β-actin (42 kDa). Protein expression of p38 MAPK or c-Jun was normalized to β-actin and is shown as the fold change relative to GAPDH siRNA-transfected cells (arbitrarily assigned as 1). The data represent mean ± SD of three independent experiments. Statistical analysis was performed using the two-tailed unpaired Student’s t-test, *p < 0.05, **p < 0.01.

As with numerous previous published studies, including those from our laboratory^7^,^12^,^13^,^18^, TGF-β inhibited the mRNA expression of CD36, SR-B1, SR-A1 and LPL in THP-1 macrophages (data not shown). In addition, where analyzed, the responses were conserved in primary HMDM and at the protein level (data not shown). We therefore decided to investigate the role of p38 MAPK and JNK pathways in the TGF-β-mediated regulation of expression of these genes using small interfering RNA (siRNA)-mediated RNA interference assays. Previous studies have shown good functional correlation between the effects of knockdown of specific proteins (e.g. Smad2/3) on the expression of these genes with the uptake of modified LDL by macrophages^7^. Comparisons were made with knockdown of glyceraldehyde-3-phosphate dehydrogenase (GAPDH), which has been used as a negative control in several previous studies^7^,^19^,^20^,^21^. Because of problems encountered with knockdown of JNK-1/2, its key downstream target, c-Jun, was included in the analysis along with p38 MAPK. The knockdown was achieved using commercially validated siRNA sequences and the relative expression following knockdown was determined by western blot analysis. As shown in Fig. 1, transfection of the cells with siRNA against p38 MAPK or c-Jun resulted in a significant knockdown of the expression of the corresponding proteins compared to control GAPDH in both vehicle treated cells (*p* = 0.010 for p38 MAPK and *p* = 0.016 for c-Jun) and those incubated with TGF-β (*p* = 0.001 for p38 MAPK and *p* = 0.031 for c-Jun). The effect of knockdown on the TGF-β-mediated regulation of expression of CD36, SR-B1, SR-A1 and LPL, which are involved in the uptake of modified lipoproteins, was next analyzed by real-time quantitative PCR (RT-qPCR). The time point of RNA isolation for these RT-qPCR was the same as that for protein used for western blot analysis, thereby providing direct correlation between knockdown and changes in gene expression. The TGF-β response following knockdown of GAPDH was compared to that following knockdown of p38 MAPK or c-Jun.

**Figure 2:**
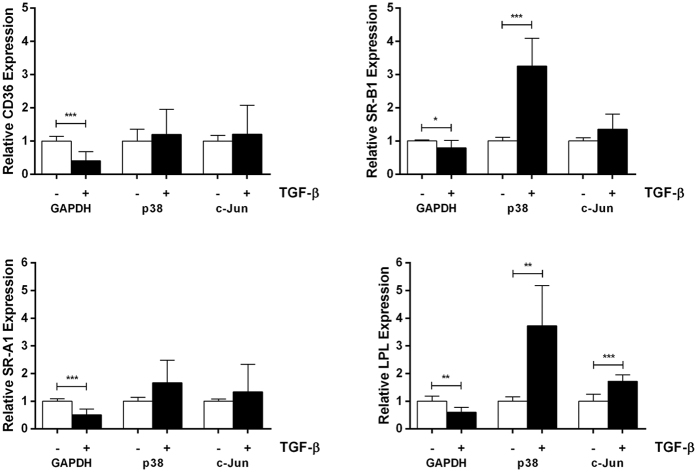
p38 MAPK and c-Jun are involved in the TGF-β-regulated expression of key cholesterol uptake genes in human macrophages. Knockdown of GAPDH or p38 MAPK or c-Jun expression in THP-1 monocytes, differentiation of monocytes into macrophages and incubation with vehicle (−, empty bars) or TGF-β (30 ng/ml) (+, filled bars) for 24 h was carried out as in Fig. 1. Total RNA was subjected to RT-qPCR using primers against CD36, SR-B1, SR-A1, LPL or RPL13A. The mRNA expression levels were determined using the comparative C_t_ method and normalized to RPL13A with the value from vehicle treated cells arbitrarily assigned as 1. The data represent mean ± SD of three independent experiments. Statistical analysis was performed using the two-tailed unpaired Student’s t-test, *p < 0.05, **p < 0.01, ***p < 0.001.

The expression of CD36, SR-B1, SR-A1 and LPL was inhibited by TGF-β following knockdown of GAPDH (*p* = 0.0009 for CD36, *p* = 0.048 for SR-B1, *p* = 0.0004 for SR-A1 and *p* = 0.003 for LPL) (Fig. 2). Thus, the previously noted TGF-β-mediated inhibition of expression of these genes in macrophages was also observed following knockdown of GAPDH. However, following knockdown of p38 MAPK, the ability of the cytokine to inhibit the expression of these genes was abolished with a significant induction of expression observed for SR-B1 (*p* = 0.00007) and LPL (*p* = 0.001) in TGF-β-treated, p38 MAPK siRNA-transfected cells (Fig. 2). Similarly, the knockdown of c-Jun attenuated the TGF-β-mediated inhibition of expression of these genes with induced levels seen with LPL mRNA (*p* = 0.0005) in the presence of the cytokine (Fig. 2). Multiple comparisons of basal expression of these genes following knockdown of GAPDH, p38 MAPK or c-Jun by one-way ANOVA with Tukey’s posthoc test revealed no significant changes (data not shown). Overall, therefore, these data suggest a critical role for both these pathways in the inhibitory action of TGF-β on the expression of these key genes implicated in macrophage uptake of modified LDL.

### The role of JNK/c-Jun signaling in the TGF-β-mediated inhibition of LPL gene transcription

**Figure 3:**
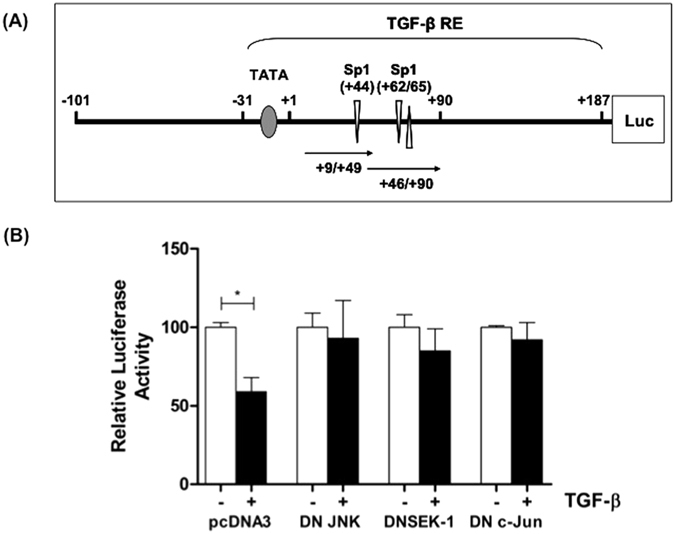
The TGF-β-mediated inhibition of minimal LPL promoter activity in human macrophages is attenuated by transfection of plasmids specifying for DN forms of JNK, SEK-1 and c-Jun. (**A**) Schematic representation of the regulatory region of the LPL gene identified in a previous study^18^. The −101 to +187 region linked to the luciferase reporter gene (Luc) is shown. The −31 to +187 sequence contains the TGF-β response elements (TGF-β RE) with three conserved Sp1/Sp3 binding sites required for the response (a single site at position +44 and a dual site at position +62/+65)^18^. The +9/+49 and +46/+90 sequences used for EMSA (Fig. 4) are also shown. (**B**) U937 cells were transfected with the minimal LPL promoter-luciferase construct (−101/+187 in the pGL2 Basic-luciferase vector) and DN JNK, DNSEK-1, DN c-Jun or pcDNA3 control vector. The cells were then differentiated with PMA (1 μM) for 12 h and then treated with vehicle (−, empty bars) or TGF-β (30 ng/ml) for further 12 h (+, filled bars). The luciferase activity was normalized to the protein concentration and is expressed as Relative Luciferase Activity. In each case, the value in cells treated with vehicle has been arbitrarily assigned as 100%. The data represent mean ± SD from three independent experiments. Statistical analysis was performed using the two-tailed unpaired Student’s t-test, *p < 0.05.

We have previously investigated the molecular mechanisms underlying the TGF-β-mediated inhibition of LPL gene transcription in more detail^18^. These studies revealed a critical role for the Sp1/Sp3-binding sites in the regulatory region of the LPL gene in the cytokine response (Fig. 3A)^18^. TGF-β had no effect on Sp1/Sp3 DNA binding but decreased its *trans*-activation potential^18^. However, the studies provided no insight into the co-activators that could potentially be involved in the TGF-β response. Our previous studies have also shown that the action of JNK and p38 MAPK converges on c-Jun/AP-1 in the TGF-β-mediated regulation of apoE expression^16^. In the light of these findings and the advanced nature of our previous studies with the availability of several reagents, we decided to investigate the role of JNK/c-Jun/AP-1 signaling in the TGF-β-mediated inhibition of LPL gene transcription in more detail.

Preliminary experiments using pharmacological inhibitors on the mouse J774.2 macrophage cell line coupled with semi-quantitative reverse transcription polymerase chain reaction (RT-PCR) showed that the TGF-β-mediated inhibition of LPL mRNA expression was attenuated by curcumin, which is known to inhibit the c-Jun/AP-1 pathway (data not shown). To investigate further the link between the JNK/c-Jun/AP-1 signaling pathway and the TGF-β-regulated LPL gene transcription, transient transfection assays were carried using the −101/+187 regulatory region of the LPL gene, which contains the three conserved Sp1/Sp3 binding sites required for the response [a single site at position +44 (antisense strand) and a dual site at position +62 (antisense strand) and +65 (sense strand)] (Fig. 3A)^18^, and expression plasmids specifying for dominant negative (DN) forms of three components of the pathway; JNK, SEK-1 and c-Jun. Unfortunately, THP-1 macrophages (and J774.2 macrophages used for a number of our previous studies) are difficult to transfect with exogenous DNA at high efficiency. The cytokine regulation of LPL gene expression is conserved in a range of macrophage cell lines and primary cultures from various species^3^,^18^,^22^,^23^,^24^,^25^ and references therein. We therefore previously tested a range of monocyte/macrophage cell lines and found that the human U937 myeloid leukemic cell line could be transfected most efficiently with DNA^22^. Indeed, these cells have been used widely to investigate the regulation of macrophage gene transcription, including our previous research on promoter dissection of LPL in relation to transcriptional regulation by interferon-γ (IFN-γ) and TGF-β^18^,^22^. All transfection assays in this study were therefore carried out using U937 cells using previously optimized conditions^18^. As shown in Fig. 3B, the significant TGF-β-mediated reduction in luciferase activity seen in cells transfected with the control pcDNA3 plasmid (*p* = 0.023) was attenuated by transfection of DN forms of JNK, SEK-1 and c-Jun, thereby lending further support to an important role of SEK-1/JNK/c-Jun in the TGF-β-mediated inhibition of LPL gene expression.

**Figure 4:**
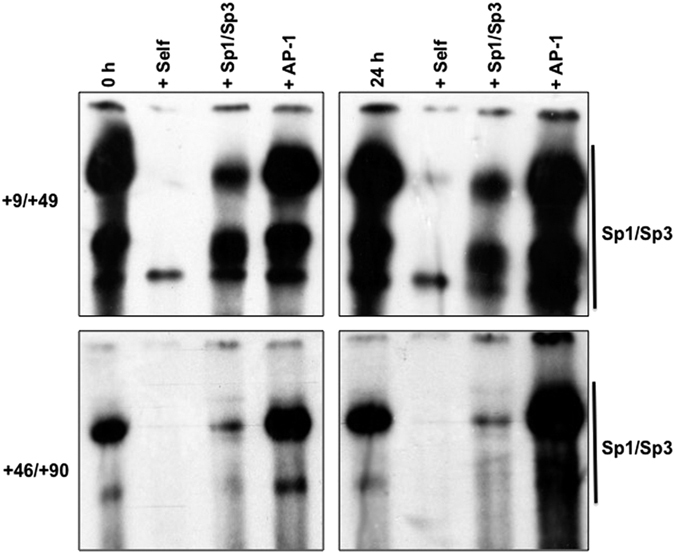
AP-1 does not interact with the TGF-β response element in the regulatory region of the LPL gene. EMSA were carried out using radiolabelled +9/+49 or +46/+90 sequence and whole cell extracts from THP-1 macrophages that were either untreated (0 h) or incubated with 30 ng/ml of TGF-β for 24 h (24 h). +Indicates competition with a 400-fold molar excess of unlabelled complementary oligonucleotide (self), Sp1/Sp3 binding site (Sp1/Sp3) or AP-1 binding site (AP-1). The DNA-protein interactions with the +9/+49 and the +46/+90 sequences have been previously characterized^18^,^22^. The region of the autoradiogram containing the Sp1/Sp3 DNA protein complexes (indicated by a vertical line) is shown. The data are representative of three independent experiments.

Because c-Jun is a key member of the AP-1 family, we next investigated by competition electrophoretic mobility shift assays (EMSA) the possibility that this transcription factor family also bound to the minimal TGF-β response elements in the regulatory region of the LPL gene. These studies employed radiolabeled oligonucleotides containing the +9/+49 and the +46/+90 regions^18^,^22^ (Fig. 3A) and extracts from untreated cells and those incubated with TGF-β for 24 h. Binding of Sp1/Sp3 to these sites was competed by an excess of oligonucleotides containing the corresponding sequence and consensus sites for Sp1/Sp3 but not that for AP-1 (Fig. 4). These studies demonstrate that AP-1 does not directly bind to the minimal TGF-β response elements in the LPL gene promoter thereby suggesting other mechanisms for the TGF-β response.

**Figure 5:**
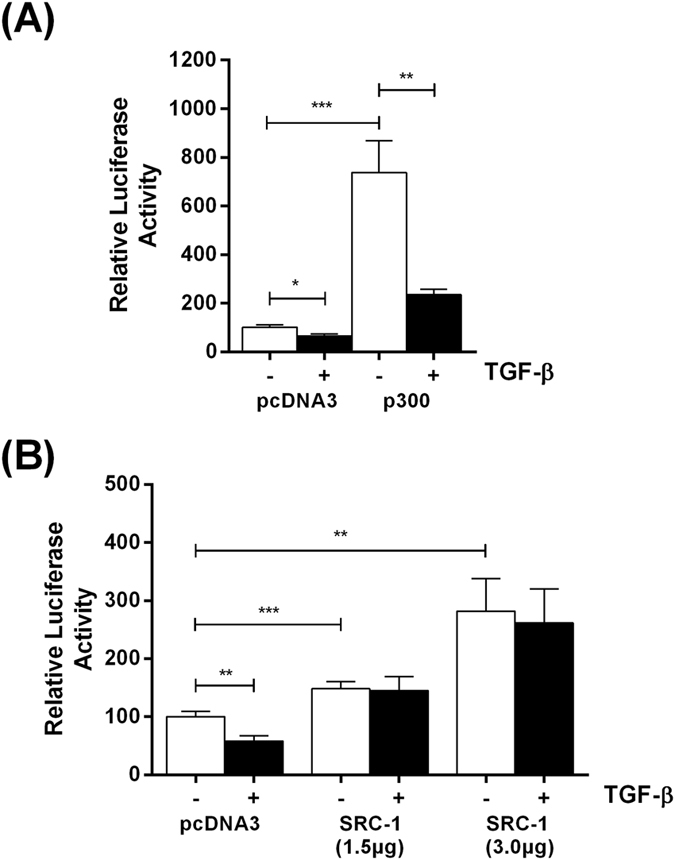
The TGF-β-mediated inhibition of minimal LPL promoter activity is attenuated by transfection of SRC-1 expression plasmid but not that for p300/CBP. U937 cells were transfected with the minimal LPL promoter-luciferase construct (−101/+187 in the pGL2 Basic-luciferase vector) and pcDNA3 control vector (pcDNA3) or p300/CBP expression plasmid (p300) (1.5 μg) **(A)** or SRC-1 expression plasmid (SRC-1) (1.5 and 3.0 μg as indicated) **(B)**. The cells were then differentiated with PMA (1 μM) for 12 h and then either treated with vehicle (−, empty bars) or TGF-β (30 ng/ml) for further 12 h (+, filled bars). The luciferase activity was normalized to the protein concentration and is expressed as Relative Luciferase Activity with the value in cells transfected with the control pcDNA3 plasmid and treated with vehicle arbitrarily assigned as 100%. The data represent mean ± SD from three independent experiments. Statistical analysis was performed using one-way ANOVA with Tukey’s post-hoc test, *p < 0.05, **p < 0.01, ***p < 0.001.

The IFN-γ-mediated inhibition of SR-A1 gene transcription is mediated through competition between Janus kinase (JAK)/signal transducer and activator of transcription (STAT) and Ras/AP-1 for a limiting amount of the co-activator p300/CREB-binding protein (CBP)^26^. Similar co-activator competition models have been identified in several settings of transcriptional inhibition^27^,^28^,^29^. We therefore wondered whether a co-activator competition model involving p300/CBP might also be applicable, at least in part, in the regulation of LPL gene transcription by this cytokine because of its involvement in transcriptional regulation by Sp1/Sp3, AP-1 or TGF-β^30^,^31^,^32^. If this were the case then the TGF-β-mediated inhibition of LPL promoter activity should be attenuated by transfection of an expression plasmid for p300/CBP. As shown in Fig. 5A, a significant inhibitory action of TGF-β on the LPL promoter activity was seen when the cells were transfected with the pcDNA3 control (*p* = 0.010) or p300/CBP (*p* = 0.004) plasmids. The co-activator was functional as it augmented the basal LPL promoter activity (*p* = 0.0002) (Fig. 5A). Sterol receptor co-activator-1 (SRC-1) has also been implicated in signaling by TGF-β^33^,^34^ and was therefore also included for comparative purposes. In contrast to p300/CBP, the TGF-β-mediated inhibition of LPL promoter activity (*p* = 0.0041) seen with the control pcDNA3 plasmid was attenuated by transfection of the cells with two concentrations of the SRC-1 expression plasmid (1.5 μg and 3.0 μg) (Fig. 5B). In addition, SRC-1 augmented the basal LPL promoter activity (*p* = 0.0000132 at 1.5 μg and *p* = 0.0054 at 3 μg) (Fig. 5B).

**Figure 6:**
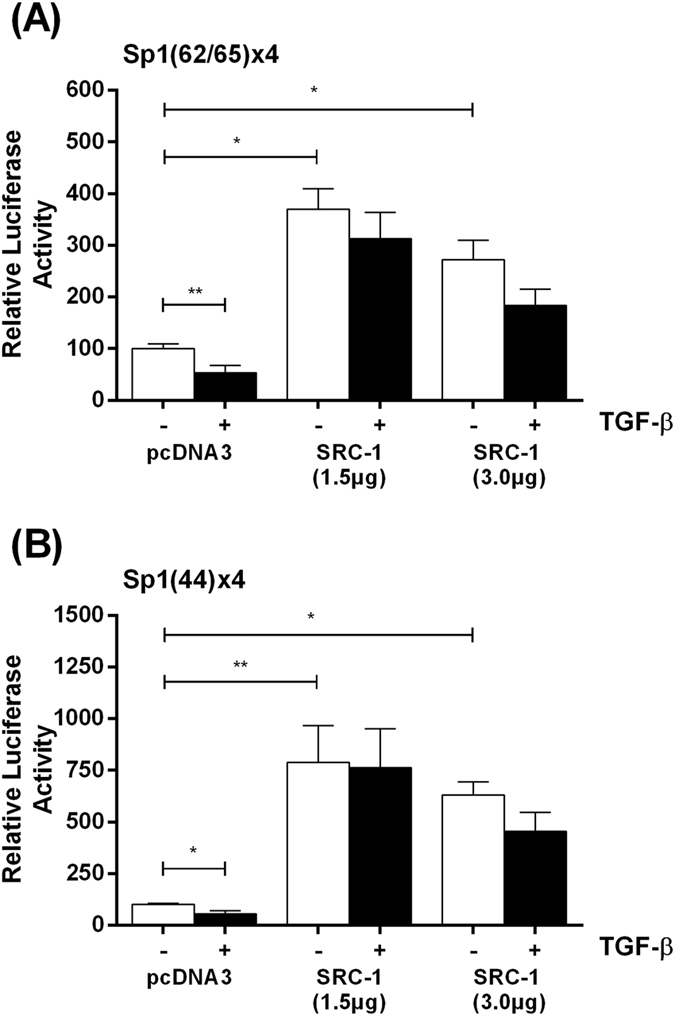
The TGF-β-mediated inhibition of activation by multiple Sp1/Sp3 sites is attenuated by transfection of SRC-1 expression plasmid. U937 cells were transfected with the an artificial promoter containing four copies of Sp1/Sp3 site at +62/+65 [Sp1 (62/65)x4] **(A)** or +44 [Sp1(44)x4 **(B)**] from the TGF-β response element in the regulatory region of the LPL gene (Fig. 3A) linked to the minimal SV40 promoter, and pcDNA3 control vector (pcDNA3) or SRC-1 expression plasmid (1.5 and 3 μg as indicated). The cells were then differentiated with PMA (1 μM) for 12 h and then incubated with vehicle (−, empty bars) or TGF-β (30 ng/ml) for further 12 h (+, filled bars). The luciferase activity was normalized to the protein concentration and is expressed as Relative Luciferase Activity with the value in cells transfected with the control pcDNA3 plasmid and treated with vehicle arbitrarily assigned as 100%. The data represent mean ± SD of three independent experiments. Statistical analysis was performed using one-way ANOVA with Tukeys post-hoc test, *p < 0.05, **p < 0.01.

We have previously shown that multimers of Sp1/Sp3 binding sites from the LPL gene can impart the TGF-β responsiveness to a heterologous minimal SV40 promoter^18^. We therefore investigated the effect of transfection of the SRC-1 expression plasmid on the reporter gene activity of DNA constructs that contained four copies of the Sp1/Sp3 binding sites from the +62/+65 region or the +44 region in front of the minimal SV40 promoter in the pGL2-Promoter vector (Fig. 6). Transfection of the SRC-1 expression plasmid augmented the basal Sp1/Sp3 promoter activity (i.e. in the absence of TGF-β) (*p* = 0.011 for 1.5 μg SRC-1 plasmid and *p* = 0.010 for 3 μg SRC-1 plasmid in the case of panel A, and *p* = 0.006 for 1.5 μg SRC-1 plasmid and *p* = 0.010 for 3 μg SRC-1 plasmid in the case of panel B). In addition, the significant TGF-β-mediated inhibition in promoter activity seen when the cells were transfected with the control pcDNA3 plasmid (*p* = 0.009 in panel A and *p* = 0.010 in panel B) was attenuated following transfection of the SRC-1 plasmid at both concentrations.

**Figure 7:**
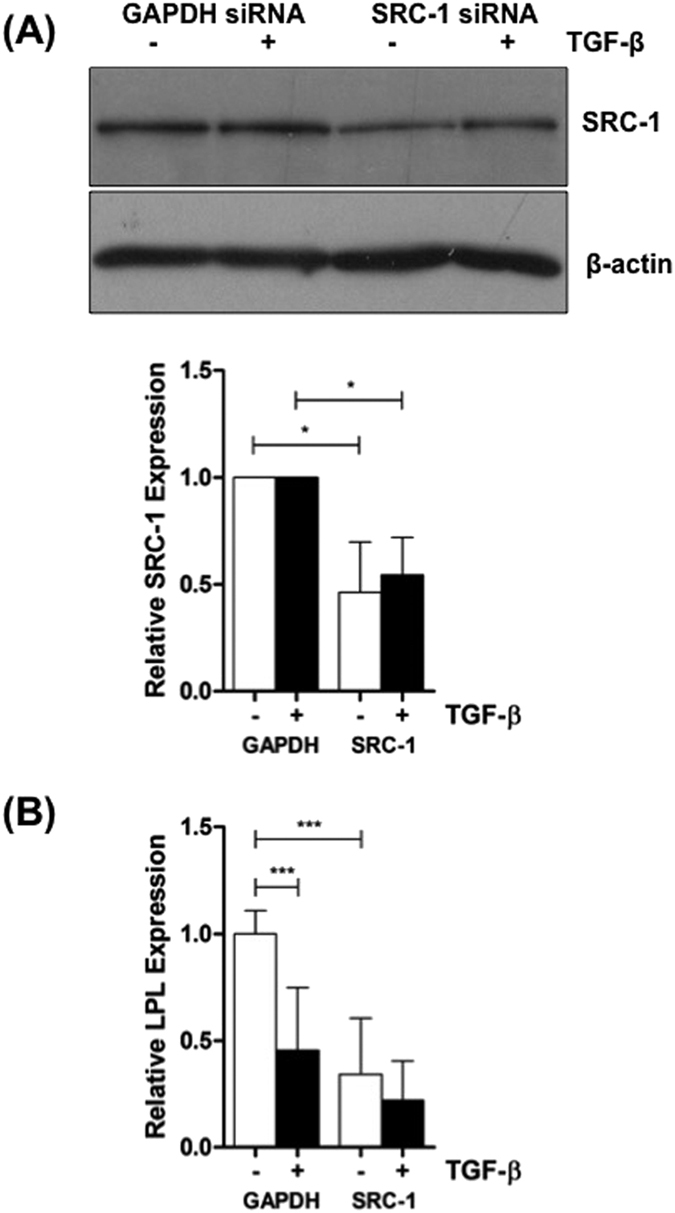
Knockdown of SRC-1 affects LPL gene expression. Knockdown of SRC-1 expression in THP-1 monocytes, differentiation of monocytes into macrophages and incubation with vehicle (−, empty bars) or TGF-β (30 ng/ml) (+, filled bars) for 24 h was carried out as in Fig. 1. (**A**) Equal amounts of protein extracts were subjected to western blot analysis using antisera against SRC-1 or β-actin. The image shows the signal from the immunoreactive SRC-1 (160 kDa) or β-actin (42 kDa). The histogram below the image shows protein expression of SRC-1 normalized to β-actin and is expressed as a fold change relative to GAPDH siRNA-transfected cells (arbitrarily assigned as 1). The data represent mean ± SD of three independent experiments. Statistical analysis was performed using the Student’s t-test, *p < 0.05. **(B)** RT-qPCR on total RNA was carried out using primers against LPL or RPL13A. The mRNA expression levels were determined using the comparative C_t_ method and normalized to RPL13A. The value in cells transfected with GAPDH siRNA and treated with vehicle was arbitrarily assigned as 1. The data represent mean ± SD of five independent experiments. Statistical analysis was performed using one-way ANOVA with Tukey’s post-hoc test, ***p < 0.001.

The transfection data suggest that SRC-1 is required for constitutive LPL expression (i.e. in the absence of TGF-β) and that transcription factors activated by the cytokine (such as c-Jun/AP-1), compete for a limiting amount of SRC-1 to contribute, at least in part, in the reduction in LPL gene expression. If this were the case then knockdown of SRC-1 should reduce constitutive LPL expression and possibly also affect the cytokine response. Knockdown experiments were therefore carried out in THP-1 macrophages to further evaluate the role of SRC-1. Western blot analysis demonstrated significant knockdown of SRC-1 by the corresponding siRNA (*p* = 0.0167 in the presence of vehicle and *p* = 0.0105 with the cytokine) (Fig. 7A). As expected, RT-qPCR showed that knockdown of SRC-1 resulted in a reduction of constitutive LPL mRNA expression (*p* = 0.00001) (Fig. 7B). In addition, the significant TGF-β-regulated inhibition of LPL expression seen in cells transfected with GAPDH siRNA (*p* = 0.0001) was attenuated (Fig. 7B).

## Discussion

The formation of lipid-laden foam cells is a critical early stage process in atherosclerosis development^1^. The anti-atherogenic cytokine TGF-β inhibits foam cell formation in part through the suppression of expression of key genes implicated in cellular cholesterol uptake^1^,^2^,^7^,^8^,^9^. The impact of MAPK pathways in the anti-atherogenic actions of TGF-β remains poorly understood. For example, although TGF-β induced apoptosis of endothelial cells via p38 MAPK^35^, this is not anti-atherogenic. In addition, growth inhibition of vascular smooth muscle cells by TGF-β required p38 MAPK^36^. However, this cannot be regarded as anti-atherogenic because the extracellular matrix produced by them is athero-protective. Although p38 MAPK and JNK are involved in TGF-β mediated inhibition of a disintegrin and metalloproteinase with thrombospondin motif-4 (ADAMTS-4) expression^21^, no evidence exists that this protease is pro-atherogenic. In this study, we show for the first time that the p38 MAPK and JNK/c-Jun signaling pathways play an integral role in the TGF-β-regulated expression of four key genes implicated in the uptake of modified LDL, CD36, SR-B1, SR-A1 and LPL. In addition, we identify an important role for the co-activator SRC-1 in the control of LPL gene expression.

Western blot analysis and nonradioactive kinase activity assays showed activation of p38 MAPK and JNK pathways in human macrophages (Supplementary Fig. 1). These findings correlate well with studies that have shown that MAPK signaling is active in macrophages and human atherosclerotic lesions^37^,^38^. We have also previously shown that TGF-β activates the JNK and p38 MAPK pathways in THP-1 monocytes^16^. The activation of p38 and JNK/c-Jun in human macrophages occurs with slower kinetics (maximal activation typically at 12–24 h) (Supplementary Fig. 1) compared to that of Smads (maximal activation typically at 30 min)^7^. Such slow kinetics suggests that MAPK activation occurs following classical TGF-β-Smad pathway activation and/or Smad-dependent transcriptional responses.

Detailed analysis of gene expression following siRNA knockdown assays revealed roles for p38 MAPK in the TGF-β-regulated inhibition of CD36, SR-B1, SR-A1 and LPL mRNA expression (Fig. 2). Previous studies have shown good correlation between the effects of knockdown of specific signaling proteins (e.g. Smad-2/3) on the expression of these genes and the uptake of modified LDL^7^. Other studies monitoring different parameters have also identified an important role for p38 MAPK in foam cell formation though it should be noted that none of these used TGF-β. For example, blockade of this pathway in murine J774 macrophages using pharmacological inhibitor SB203580 showed its requirement in oxLDL-induced CD36 expression and subsequent foam cell formation through transactivation of peroxisome proliferator-activated receptor-γ (PPARγ)^39^. The use of pharmacological inhibitors also demonstrated a requirement of p38 MAPK in the promotion of foam cell formation by iron deficiency, though this was independent of PPARγ^40^. Our studies extend these findings via the use of siRNA-mediated knockdown assays, which are more specific than the use of pharmacological agents, and the expression of key genes involved in cellular cholesterol uptake. Other processes, such as macropinocytosis and macroautophagy are also involved in foam cell formation^41^,^42^. It is interesting that p38 MAPK is also involved in the promotion of cholesteryl ester accumulation in macrophages via the inhibition of macroautophagy^41^. However, previous studies investigating the role of p38 MAPK *in vivo* have not yielded consistent outcomes^43^,^44^. The precise reasons for such discrepancies are currently unclear but direct correlations with this study cannot be made as none of them involved TGF-β or focussed specifically on macrophage foam cell formation and the expression of key genes implicated in the uptake of modified LDL. In addition, functional redundancy could have made some contribution given that at least four isoforms of p38 MAPK exist^2^,^38^.

Knockdown of c-Jun expression also demonstrated a requirement for this JNK-activated transcription factor in the TGF-β-regulated expression of CD36, SR-B1, SR-A1 and LPL (Fig. 2). The JNK signaling pathway has previously been suggested to have a pro-atherogenic role in foam cell formation *in vivo* though again this did not involve TGF-β^37^. Thus, JNK2^−/−^ApoE^−/−^ but not JNK1^−/−^ApoE^−/−^ mice were found to be resistant to diet-induced atherosclerosis^37^. In addition, treatment of macrophages from CD36^−/−^ mice with a JNK inhibitor blocked oxLDL-induced foam cell formation^45^. Our study demonstrates that the JNK signal transduction pathway also plays a crucial role in the negative regulation of foam cell formation by TGF-β at least at the level of expression of key genes involved in the uptake of modified lipoproteins.

We also investigated the role of JNK/c-Jun signaling in the TGF-β-mediated inhibition of gene expression involved in modified lipoprotein uptake via further studies on LPL. Transfection assays, EMSA and siRNA-mediated knockdown studies (Figs 3–7) suggest that competition between Sp1/Sp3 and c-Jun/AP-1 (or other TGF-β activated transcription factors), for a limiting amount of SRC-1 is likely to play a major role. In this model, SRC-1 is required for constitutive LPL expression via Sp1/Sp3. This proposition is supported by increased activity of minimal LPL promoter containing the TGF-β response elements or multiple copies of Sp1/Sp3 sites linked to a heterologous promoter following transfection of the cells with an SRC-1 expression plasmid (Figs 5 and 6) together with reduced constitutive levels of LPL mRNA following knockdown of SRC-1 expression (Fig. 7). The TGF-β-mediated activation of c-Jun/AP-1 (and potentially other factors), which does not interact with the cytokine response elements in the minimal promoter region (Fig. 4), is likely to reduce the amount of SRC-1 available for transactivation of the LPL gene by Sp1/Sp3. Indeed, the TGF-β-mediated reduction in promoter activity observed with the minimal LPL promoter containing the TGF-β response elements or multiple copies of Sp1/Sp3 sites linked to a heterologous promoter was attenuated following transfection of the cells with an SRC-1 expression plasmid (Figs 5 and 6). SRC-1 has been shown to interact with c-Jun and c-Fos subunits and regulate AP-1-mediated transactivations^46^. The binding of SRC-1 to c-Jun and c-Fos was demonstrated by glutathione S-transferase pull down assays and the yeast two-hybrid system^46^. The interaction sites were mapped to a region of SRC-1 that contains strong intrinsic histone aceytltransferase activity^46^. Interestingly, the IFN-γ-mediated inhibition of SR-A1 transcription is mediated by competition between STAT1 and AP1/Ets for a limiting amount of p300/CBP^26^. Our studies suggest that such models extend to SRC-1. This co-activator has been shown to potentiate TGF-β/Smad signalling via a mechanism that involves p300/CBP^33^. The studies here suggest additional involvement of SRC-1 in the inhibition of gene expression that is independent of p300/CBP.

We have previously shown that the classical TGF-β signal transducers, Smad-2 and Smad-3 have crucial roles in the regulation of gene expression and the uptake of modified lipoproteins by this cytokine^7^. The genes included SR-A1, LPL and CD36 with Smad-2 playing a more dominant role^7^. The studies presented here show an important role for p38 MAPK and JNK/c-Jun in the TGF-β-mediated inhibition of expression of these genes. Crosstalk between signaling pathways is relatively common and many studies have demonstrated an involvement for the classical TGF-β-activated Smad pathway and one or more of the MAPK pathways in the regulation of gene expression by this cytokine. For example, c-Jun and Smad-3 are able to interact in the regulation of gene expression^47^,^48^,^49^. Our studies suggest that such cross talk is likely to extend to the control of macrophage cholesterol homeostasis and associated changes in gene expression. It is unlikely that direct binding by Smads is involved, at least in the case of LPL, as promoter dissection and DNA protein interaction studies have identified an important role for Sp1/Sp3^18^. Further studies should seek to delineate the mechanisms underlying such cross-talk, particularly the functional interactions between the Smads and MAPK along with various other components involved in signaling, together with the relative contribution of each pathways in the TGF-β-mediated regulation of genes implicated in macrophage uptake of modified LDL and foam cell formation both in macrophages and in mouse models of atherosclerosis. Given the different kinetics in the activation of Smads and MAPK^7^ (Supplementary Fig. 1), it is possible that the latter are involved in more prolonged changes in gene expression and cellular changes mediated by this cytokine, such as foam cell formation and genes implicated in its regulation. It should also be noted that not all pathways for macrophage foam cell formation require Smads. For example, Smads were not involved in the TGF-β-mediated inhibition of macropinocytosis^42^.

In conclusion, we have demonstrated for the first time that the MAPK p38 and JNK/c-Jun play crucial roles in the TGF-β-mediated regulation of expression of key genes involved in macrophage cholesterol homeostasis. In addition, we have identified a potential mechanism for the regulation of LPL gene expression. Future studies should analyse the promoter regions of SR-A1, SR-B1 and CD36 in order to identify TGF-β response element(s) and the transcription factors that interact with such elements together with the role of SRC-1 in transcriptional inhibition of these genes via approaches described here. Such studies are important for atherosclerosis given the substantial anti-atherogenic action of TGF-β and the potential for therapies targeted against foam cell formation and atherosclerosis development.

## Materials and Methods

### Reagents

Human recombinant TGF-β1 was supplied by Peprotech (London, UK). Validated c-Jun and SRC-1 (NCOA-1) small interfering RNA (siRNA) were from Qiagen (SI00300580 and SI00055342 respectively) (Crawley, UK) and validated p38 MAPK and GAPDH siRNA were from Invitrogen (Paisley, UK). Validated antibodies were purchased from Cell Signaling Technology (Herfordshire, UK) [JNK, phospho JNK (Thr 183/Tyr 185), p38MAPK, phospho p38MAPK (Thr 180/Tyr 182), SEK-1 and phospho SEK-1 (Ser 257/Thr 261)], Santa Cruz Biotechnology (c-Jun and SRC-1) (California, USA) and Sigma-Alrich (Poole, UK) (β-actin).

### Cell culture

HMDMs were differentiated from monocytes isolated from buffy coats (Welsh Blood Service, Pontyclun UK) using Ficoll-Hypaque purification as described elsewhere^5^,^6^,^7^. Ethical approval and informed consent for each donor was granted by the Welsh Blood Service for the use of human blood samples. The human monocytic THP-1 and U937 cell lines together with HMDM were maintained in complete RPMI 1640 medium supplemented with 10% (v/v) (THP-1 or U937) or 5% (v/v) (HMDM) heat-inactivated fetal calf serum, 100 U/ml penicillin, and 100 μg/ml streptomycin. All cell cultures were maintained at 37 °C in a humidified atmosphere containing 5% (v/v) CO_2_ in air. Differentiation of THP-1 monocytes into macrophages was performed for 24 h using 0.16 μM phorbol 12-myristate 13-acetate (PMA).

### Western blot analysis and kinase activity assays

Whole-cell extracts were prepared in buffers containing phosphatase and protease inhibitors and used for Western blot analysis as previously described^16^,^50^,^51^. The proteins were subjected to electrophoresis alongside comparative molecular weight markers (GE Healthcare) to determine the size of the protein product. Non-radioactive JNK activity assays were carried out using kits from Cell Signaling Technology according to the manufacturer’s instructions.

### Real-time quantitative PCR (RT-qPCR)

This was performed using SYBR Green JumpStart *Taq* ReadyMix (Sigma-Aldrich) and the Opticon 2 RT-qPCR detection system (MJ Research)^5^,^6^,^7^ (see Supplementary Table 1 for sequences of primers). The mRNA levels were determined using the comparative C_*T*_ method and normalized to the ribosomal protein L13A (RPL13A) mRNA levels^5^,^6^,^7^.

### Transfection of siRNA

THP-1 monocytes were transfected with validated siRNA at a final concentration of 7.5 nM using INTERFERin^TM^ as described by the manufacturer (PolyPlus Transfection) (Nottingham, UK). The cells were then incubated for 24 h before differentiation into macrophages using PMA as described above, and subsequent treatment with TGF-β (30 ng/ml) or vehicle for 24 h. Total cellular proteins were isolated for determining knockdown by western blot analysis and total RNA for gene expression studies.

### Transient transfection and electrophoretic mobility shift assays (EMSA)

Transient transfection of U937 cells with an LPL promoter-luciferase DNA construct (−101 to +187)^18^,^22^,^23^ or multimers of Sp1-binding site in the LPL promoter upstream of a minimal SV40 promoter in the pGL2-promoter vector^18^,^22^ was carried out using SuperFect^TM^ (Qiagen) as previously described^18^,^22^,^23^. EMSA with whole cell extracts was essentially carried out as previously reported^18^,^22^,^23^. In competition assays, the binding mixture was incubated for 10 min on ice with a 400-fold molar excess of unlabelled competitor oligonucleotides prior to the addition of the radiolabelled probe. The sequences of the oligonucleotides were: +9/+49, 5′-CTCGATTTCTCCTCCTACTCCTCCTCCGAGGAATTCT-3′ and 5′-GGGCAGAATTCCTCGGAGGAGGAGTAGGAGGAGAAAT-3′; +46/+90, 5′-GCCCCCTGTAACTGTTCTGCCCTCCCCTTTAAAGGTTGACTT-3′ and 5′-GGCAAGTCAACCTTTAAAGGGGAGGGCAGAACAGTTACAGGG-3′; Sp1/Sp3, 5′-TAGATTCGATCGGGGCGGGGCGAG-3′ and 5′-GCCCTCGCCCCGCCCCGATCGAAT-3′; AP-1, 5′-GATCCTTCGTGACTCAGCGGGATCCTTCGTGACT-3′ and 5′-CCGCTGAGTCACGAAGGATCCCGCTGAGTCACGAA-3′.

### Statistical analysis of data

Data are presented as mean ± SD from the numbers of independent experiments indicated in figure legends. A two-tailed unpaired Student’s t-test was used for single comparisons and a one-way ANOVA was used with Tukey’s post hoc test for multiple comparisons.

## Additional Information

**How to cite this article**: Salter, R. C. *et al.* The role of mitogen-activated protein kinases and sterol receptor coactivator-1 in TGF-β-regulated expression of genes implicated in macrophage cholesterol uptake. *Sci. Rep.* **6**, 34368; doi: 10.1038/srep34368 (2016).

## Supplementary Material

Supplementary Information
