# Supplementary material for: The role of mitogen-activated protein kinases and sterol receptor coactivator-1 in TGF-β-regulated expression of genes implicated in macrophage cholesterol uptake
Source: Sci Rep. 2016 Sep 30;6:34368. doi: 10.1038/srep34368 (PMC5043369; doi:10.1038/srep34368)
Supplement: Supplementary Information [file srep34368-s1.pdf]

**The role of mitogen-activated protein kinases and sterol receptor coactivator-1 in TGF-β-regulated expression of genes implicated in macrophage cholesterol uptake**

Rebecca C. Salter[1], Pelagia Foka[1§], Thomas S. Davies, Hayley Gallagher, Daryn R. Michael, Tim G. Ashlin and Dipak P. Ramji[*]

Cardiff School of Biosciences, Cardiff University, Sir Martin Evans Building, Museum Avenue, Cardiff, CF10 3AX, United Kingdom.

[1]: Joint first authors

[*]Corresponding Author: Dr Dipak P. Ramji, Cardiff School of Biosciences, Cardiff University, Sir Martin Evans Building, Museum Avenue, Cardiff, CF10 3AX. Phone: +44 2920 876753; Fax: +44 2920 874116; Email: Ramji@Cardiff.ac.uk

[§] Present Address: Hellenic Pasteur Institute, Molecular Virology Laboratory, 127 V. Sofias Avenue, 115-21 Athens, Greece

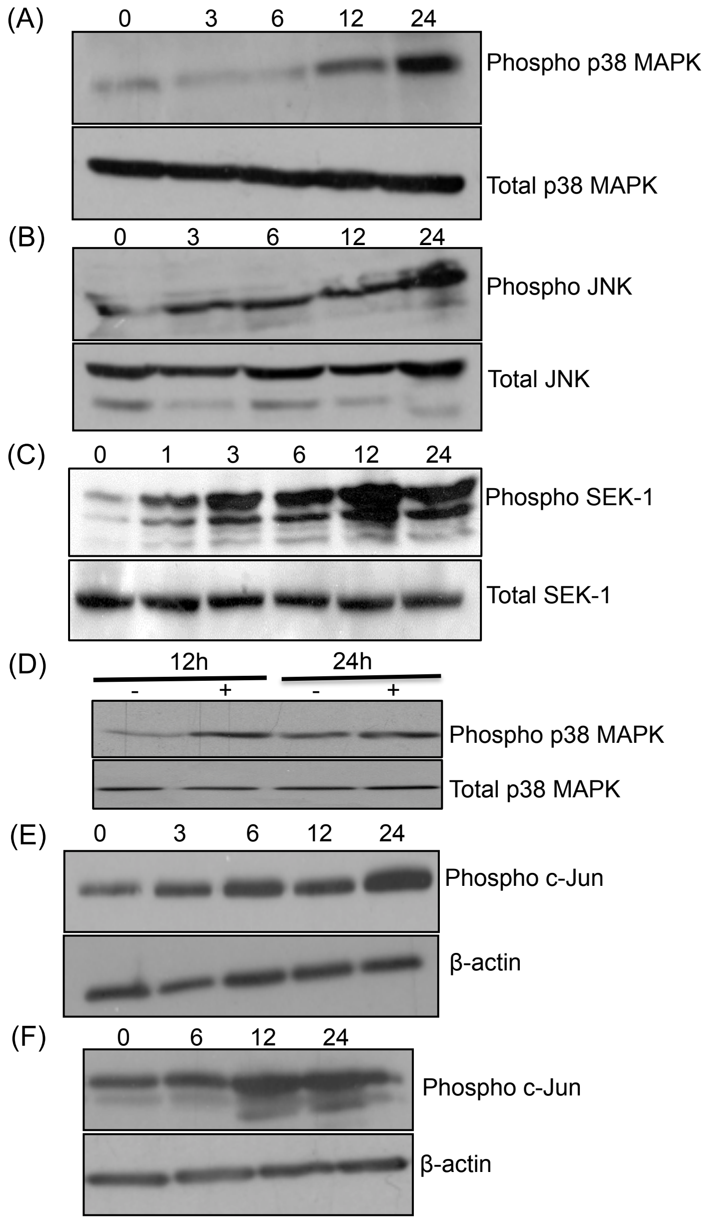


**Supplementary Figure 1. TGF-β activates p38 MAPK and JNK pathways in human macrophages**

(A-C) THP-1 macrophages were either untreated (0) or incubated with TGF-β (30 ng/ml) for the indicated period of time (hours). Equal amounts of proteins were then subjected to western blot analysis and levels of phospho-p38 MAPK (A), JNK (B) and SEK-1 (C) were compared with total levels of these proteins. (D) HMDM were incubated with vehicle (-) or TGF-β (30 ng/ml, +) for 12h or 24h as indicated. Western blot analysis was performed using antibodies against phospho-p38 MAPK or total p38 MAPK as shown. (E-F) THP-1 macrophages (E) or HMDM (F) were either untreated (0) or incubated with TGF-β (30 ng/ml) for the indicated period of time (hours). JNK in cell extracts was immunoprecipitated and *in vitro* kinase assays were carried out using recombinant c-Jun protein. The activation was monitored by western blot analysis using phospho-c-Jun antibody. Whole cell extracts were also probed with a β-actin antibody as a control for amount of protein. The images shown are representative of between two and four independent experiments.

**Supplementary Table 1: Sequence of primers used for PCR reactions**

| Gene | Sequence of forward primer (5' ➔3') | Sequence of reverse primer (5' ➔3') | Ref. |
|---|---|---|---|
| SR-A1 | CCAGGGACATGGAATGCAA | CCAGTGGGACCTCGATCTCC | 1 |
| SR-B1 | ACGACACCGTGTCCTTCC | CGGGCTGTAGAACTCCAGCGA | 2 |
| CD36 | GAGAACTGTTATGGGGCTAT | TTCAACTGGAGAGGCAAAGG | 1 |
| LPL | GAGATTTCTCTGTATGGCACC | CTGCAAATGAGACACTTTCTC | 3 |
| RPL13A | CCTGGAGGAGAAGAGGAAAGAGA | TTGAGGACCTCTGTGTATTTGTCAA | 4 |

1. Draude, G. & Lorenz, R. L. TGF-β1 downregulates CD36 and scavenger receptor A but upregulates LOX-1 in human macrophages. *Am. J. Physiol. Heart Circ. Physiol.* **278**, H1042-H1048 (2000).
2. Eguchi, A., Murakami, A. & Ohigashi, H. Nobiletin, a citrus flavonoid, suppresses phorbol ester-induced expression of multiple scavenger receptor genesin THP-1 human monocytic cells. *FEBS Lett.* **580**, 3321-3328 (2006).
3. Irvine, S. A., Foka, P., Rogers, S. A., Mead, J. R. & Ramji, D. P. A critical role for the Sp1-binding sites in the transforming growth factor-beta-mediated inhibition of lipoprotein lipase gene expression in macrophages. *Nucleic Acids Res.* **33**, 1423-1434 (2005).
4. Michael, D. R., Salter, R. C. & Ramji, D. P. TGF-β inhibits the uptake of modified low density lipoprotein by human macrophages through a Smad-dependent pathway: a dominant role for Smad2. Biochim. Biophys. Acta **1822**, 1608-1616 (2012).
